# Development of an explainable machine learning model for 3-year cardiovascular risk prediction in new-onset type 2 diabetes using the TyG index and ultrasound features

**DOI:** 10.1186/s12911-025-03247-6

**Published:** 2025-11-04

**Authors:** Zhen-zhen Jiang, Yan-feng Jiang, Yue Wang, Ying Zhou, Rong-li Peng, Cai-ye Ma, Xia-tian Liu

**Affiliations:** Department of Ultrasound, Shaoxing People`s Hospital (The First Affiliated Hospital, Shaoxing University), 568 N Zhongxing Rd, Shaoxing, Zhejiang 312000 China

**Keywords:** Cardiovascular disease, Type 2 diabetes, Machine learning, SHAP, TyG index, Ultrasound imaging

## Abstract

**Background:**

New-onset type 2 diabetes (T2D) is associated with increased cardiovascular risk and requires tailored prevention strategies. Traditional risk factors and assessment tools may not accurately predict cardiovascular disease (CVD) in this population. In our study, we compared different machine learning (ML) methods to predict the 3-year risk of developing CVD in new-onset T2D patients and developed models combining clinical data and ultrasound features for better risk evaluation.

**Methods:**

A group of 3,358 hospitalized T2D patients was screened. ML models were developed and evaluated. Feature selection was conducted via SHapley Additive exPlanations (SHAPs) and recursive feature elimination to improve both the model’s performance and its interpretability. The optimal model was subsequently compared with the Framingham Risk Score (FRS). Ultimately, the model was employed for risk stratification.

**Results:**

Of the ML models developed, LightGBM, which incorporates six features—namely, hypertension, age, the triglyceride-glucose (TyG) index, plaque burden, maximum plaque thickness, and intima-media thickness, achieved robust performance (AUC 0.845 in the training cohort and 0.772 in the validation cohort). The model outperformed the traditional FRS (AUC 0.672 in the training cohort and 0.608 in the validation cohort, *P* < 0.05). SHAP analysis enabled individualized interpretability and clinical insights. A web-based tool was deployed to facilitate clinical application.

**Conclusions:**

The predictive model developed in this study by integrating clinical and imaging data, with a focus on the TyG index and ultrasound features, demonstrated enhanced predictive capability for CVD incidence in individuals with new–onset T2D. It also allows easy risk classification and is available as a web tool for real-time use, helping improve early detection and personalized care.

**Supplementary Information:**

The online version contains supplementary material available at 10.1186/s12911-025-03247-6.

## Introduction

Diabetes mellitus, a chronic condition marked by elevated blood sugar, poses a significant global health challenge [1]. Over half of all fatalities related to diabetes are attributed to cardiovascular disease (CVD) [2]. Notably, patients with new-onset type 2 diabetes (T2D) already carry a significantly elevated risk of cardiovascular complications, even at the early stages of the disease [3]. However, because these individuals may present with relatively mild metabolic disturbances and lack overt clinical symptoms, the urgency of CVD prevention is often underestimated, leading to missed opportunities for timely intervention [4]. Early identification of high-risk patients is therefore essential, as it enables the implementation of targeted preventive strategies that can substantially reduce adverse cardiovascular outcomes [5]. In this context, developing efficient and clinically applicable tools for cardiovascular risk stratification in newly diagnosed T2D populations is imperative for mitigating long-term morbidity and mortality [6].

Traditionally, cardiovascular risk prediction has relied on conventional factors such as hypertension, dyslipidemia, obesity, and smoking status. Although valuable, these factors show only moderate predictive power, and their discriminatory ability is limited in diabetes populations [7]. Cardiovascular risk evaluation tools, such as the Systematic Coronary Risk Evaluation Score (SCORE) or the Framingham Risk Score (FRS), offer estimations of CVD risk by integrating clinical and laboratory data [8]. However, objective indicators of arteriosclerosis, such as imaging data obtained via ultrasound or CT, have not been incorporated into the development of those tools [9]. Incorporating such objective imaging indicators into risk prediction frameworks may substantially improve the accuracy and clinical utility of cardiovascular risk stratification [10], especially in individuals with newly diagnosed T2D.

Machine learning (ML) techniques have revolutionized medical research by integrating a wide range of clinical variables, including biochemical markers, demographic characteristics, and imaging features obtained from multiple imaging modalities [11–13]. However, previous ML prediction models for CVD primarily incorporate computed tomography angiography image data, which involve radiation exposure and the administration of contrast agents, restricting their clinical utility [14, 15]. In contrast, carotid ultrasound imaging offers a radiation-free and sensitive window for assessing systemic atherosclerotic conditions [16], making it a more suitable option for clinical applications [17]. Meanwhile, the triglyceride-glucose (TyG) index, which integrates both triglyceride and glucose levels and reflects the degree of insulin resistance and abnormal fat metabolism in diabetic patients, has been reported to be a valuable tool for assessing cardiovascular risk in diabetic patients by capturing the interplay between abnormal fat metabolism, insulin resistance, and dysregulated glucose metabolism [18]. Nevertheless, the effectiveness of ML algorithms that incorporate the TyG index and ultrasound features for identifying individuals with new–onset T2D who are at risk of developing CVD has yet to be established.

Therefore, we aimed to develop ML methods to assess the 3-year risk of CVD in new–onset T2D patients. Through the construction of predictive models that integrate clinical and imaging data, we hope to optimize risk evaluation, diagnostic precision, and treatment planning for this population, thereby enhancing overall outcomes and utilizing CVD risk stratification tools in routine clinical settings.

## Methods

### Study population and definitions

**Fig. 1:**
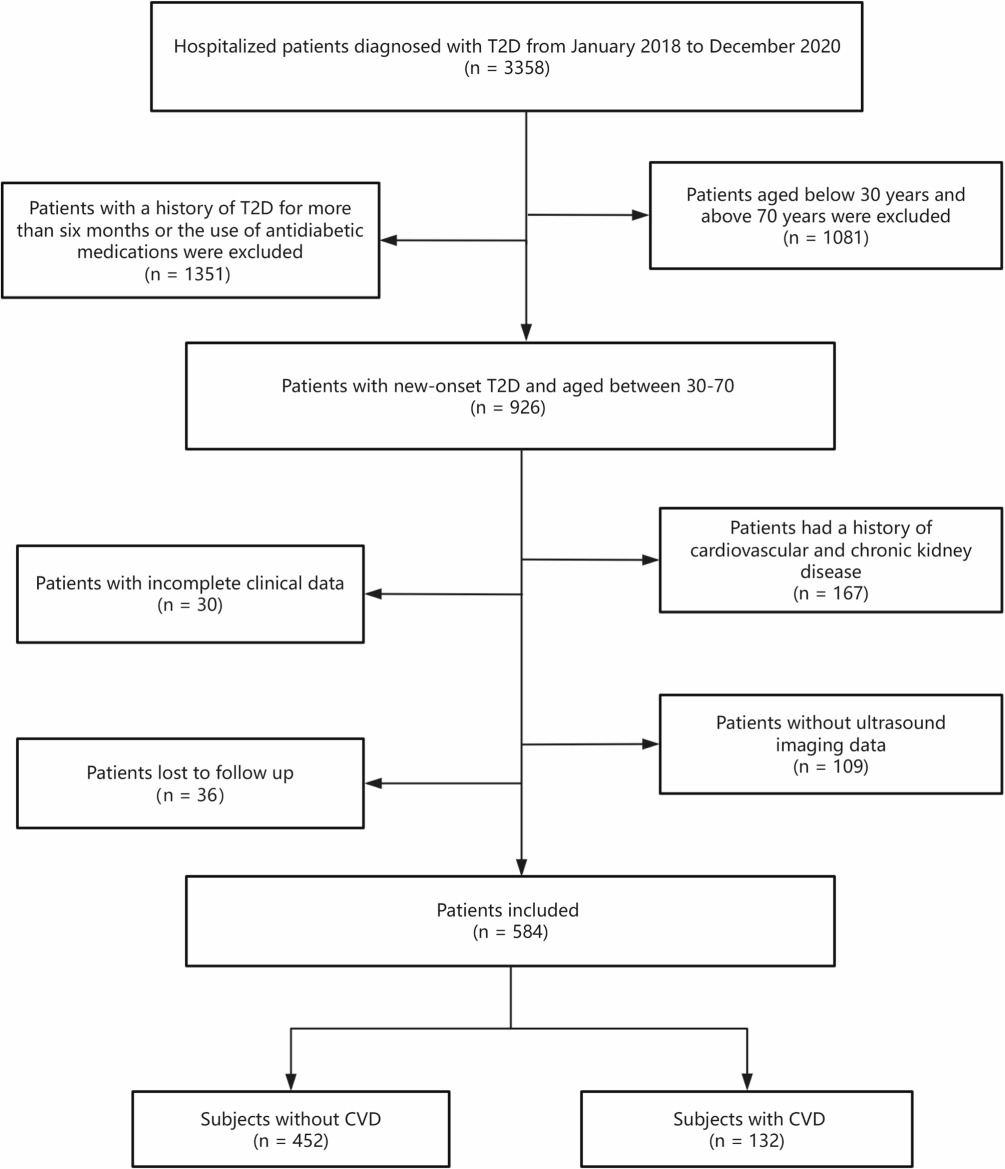
Flow chart for patient screening. T2D, type 2 diabetes; CVD, cardiovascular disease

A total of 3,358 in–hospital patients diagnosed with T2D were screened from January 2018 to December 2020. Subjects who were aged 30 to 70 years and had new-onset diabetes were considered for inclusion. Subjects who had cardiovascular or chronic kidney disease, who had incomplete data or who lacked ultrasound imaging data were excluded. Figure 1 shows the flowchart of patient selection.

New–onset T2D is diagnosed when fasting plasma glucose (FPG) concentrations exceed 126 mg/dL and haemoglobin A1c (HbA1c) values exceed 6.5% for a period of less than six months without a previous history of diabetes, according to the American Diabetes Association (ADA) Standards of Medical Care in Diabetes [19]. The term chronic kidney disease refers to glomerular filtration rates less than 30 mL/min.

### Study outcomes

The endpoint of the study was the incidence of CVD over a three-year follow-up period. CVD is a collective term for conditions affecting the heart and blood vessels, including coronary artery disease, heart failure, arrhythmias, and stroke [20].

### Data collection

Various demographic and clinical variables were incorporated for model development. Body mass index (BMI) was determined via the formula of weight divided by height squared. The TyG index was determined via the following formula: TyG index = ln (fasting triglyceride concentration multiplied by fasting blood glucose concentration divided by 2). The cardiometabolic index (CMI) was calculated as follow: CMI = (triglycerides/HDL cholesterol) × (waist circumference/height) [21]. Follow–ups through in–person visits were conducted for three years. The occurrence of CVD was verified based on clinical presentation, imaging data, or other relevant information and confirmed by specialized cardiologists or neurologists.

Ultrasound features were obtained from carotid ultrasound images. By reviewing the images, the ultrasound characteristics of the arteries, including the intima–media thickness (IMT), presence of plaque, plaque stability, maximum plaque thickness, and plaque burden, were recorded. The definition of intimal–media thickening was an IMT measurement exceeding 1.0 mm, whereas the presence of a plaque was indicated by a local IMT measurement exceeding 1.5 mm or exhibiting a 50% increase in thickness compared with adjacent tissue. The plaque burden was calculated according to the literature [3]. In brief, the patient’s bilateral carotid arteries were anatomically segmented into distinct regions, including the common, bifurcated, internal and external regions. Regions were rated on a scale of 1 to 3 based on the number of plaques or presence of stenosis. The cumulative score of these eight regions was indicative of the overall carotid plaque burden. Two experienced sonographers who were blinded to the outcomes independently reviewed the ultrasound features. If there were differences between the ultrasound results, they were determined by consensus.

### Data preprocessing

Features with a missing data percentage of no more than 20% were retained. The proportion of missing values and imputation methods were provided in Supplemental Table S1. To address potential multicollinearity among predictors, a two-step approach was employed. First, Spearman correlation analysis was performed to preliminarily identify pairs of features with high correlation coefficients. Subsequently, Variance Inflation Factor (VIF) analysis was conducted to quantitatively evaluate collinearity. Variables with a VIF greater than 10 were considered to exhibit severe multicollinearity and were excluded from further modeling. Finally, a combination of recursive feature elimination and SHapley Additive exPlanations (SHAP) was employed to comprehensively evaluate and select the features.

### Model development and comparison

ML algorithms, including random forest (RF), eXtreme gradient boosting (XGBoost), categorical boosting (CatBoost), and light gradient boosting machine (LightGBM), were used to predict the 3-year risk of cardiovascular disease in patients with new-onset type 2 diabetes. Stratified random sampling split the patients into a 70% training cohort and a 30% validation cohort based on whether the endpoint occurrence. Hyperparameters were optimized using five-fold cross-validation and grid search. To evaluate the impact of threshold selection, a sensitivity analysis was conducted across thresholds ranging from 0.1 to 0.5. The models were compared in terms of various evaluation metrics, including the area under the curve (AUC), accuracy, precision, F1 score, sensitivity, specificity, positive predictive value (PPV), and negative predictive value (NPV). Model performances were then assessed and compared on the validation cohort.

### Model interpretation

To enhance model interpretability, SHAP value was applied. SHAP values provide a systematic approach to quantifying feature importance, offering both global interpretability and local interpretability. The global explanation identifies key contributors to the model’s predictions by assigning consistent attribution values, whereas the local explanation allows for individualized predictions by analysing specific input cases. Additionally, to assess the clinical applicability of the final model, a comparative analysis was conducted against the FRS, a well-established tool for evaluating CVD risk within the general population [22]. The AUC values were plotted to assess model performance. Metrics such as accuracy and precision were also computed to quantify the performance of both models. Decision curve analysis (DCA) was utilized to examine threshold probabilities. The qualitative assessment of model calibration was conducted via a calibration curve. Additionally, Kaplan–Meier analysis was employed to evaluate the event-free survival associated with the final model.

### Web application for clinical utility

To facilitate the integration of model implementation into clinical practice, we developed an intuitive web interface utilizing Python’s Streamlit framework to incorporate the optimized risk prediction algorithm. This application allows clinicians to input patient-specific ultrasound and clinical parameters from new–onset T2D cases, subsequently enabling the system to automatically calculate the individualized 3-year CVD risk probability. To enhance interpretability, the platform generates a force plot that quantifies the influence of each predictor variable on the final risk evaluation, thereby supporting evidence-based clinical decision-making. Furthermore, the application offers tiered recommendations based on risk stratification. The implementation code is publicly available on GitHub (https://github.com/echhoc/CVD-risk-prediction-LightGBM) to facilitate reproducibility.

### Statistical analysis

The statistical analyses were conducted with SPSS (version 26.0), R (version 4.0.2), and Python (version 3.6.5). Patients were grouped into CVD and non-CVD categories according to their follow-up records. Data with skewed distributions are represented by medians and interquartile ranges, whereas data with normal distribution are shown as the means ± standard deviations. Categorical variables are expressed in numbers and percentages. Group comparisons for continuous variables were performed using t tests, and chi-square tests were utilized for categorical variables. By using the DeLong test, the AUCs of the different models were compared. A two–tailed *P* value < 0.05 was regarded as statistically significant.

## Results

### Baseline information of the patients

Table 1 presents the baseline data of the included patients. Overall, of the 584 patients analysed, 383 (65.6%) were male, and the median age was 54.00 (48.00–60.00) years. More than 44.9% of the participants had hypertension, while almost 42.3% were current smokers, and 35.8% were currently consuming alcohol. Additionally, patients with CVD were older [56.00 (51.00–65.00) vs. 53.00 (47.00–59.00) years, *P* < 0.001], more likely to be smokers (50.00% vs. 40.00%, *P* = 0.042), and more likely to have hypertension (59.10% vs. 40.70%, *P* < 0.001) than patients without CVD were. The validation cohort comprised 176 patients, including 40 CVD and 136 non-CVD cases. In terms of laboratory indicators, individuals with CVD presented notably elevated levels of total cholesterol, triglycerides, FBG, CRP, TyG index, and CMI, as well as reduced levels of HDL.

**Table 1:** Baseline data and characteristics of our study population

Variables	Overall (*n* = 584)	Non – CVD Group (*n* = 452)	CVD Group (*n* = 132)	*P* value
Age, years	54.00 (48.00–60.00)	53.00 (47.00–59.00)	56.00 (51.00–65.00)	<0.001^**^
Waist circumference, cm	88.00 (82.00–94.00)	88.00 (82.00–94.00)	89.00 (83.00–96.88)	0.108
Weight, kg	67.20 (59.13–75.88)	66.85 (58.55–75.78)	68.00 (60.90–77.35)	0.139
Height, cm	165.75 (158.50–171.38)	165.00 (158.00–171.00)	167.50 (161.13–171.50)	0.076
BMI, kg/m^2^	24.70 (22.40–26.90)	24.80 (22.33–26.80)	24.55 (22.83–27.48)	0.334
SBP, mmHg	131.00 (119.00–143.00)	130.00 (119.00–142.00)	135 (121.25–147.75)	0.064
DBP, mmHg	83.00 (76.00–90.00)	83.00 (76.00–90.00)	84.00 (76.00–91.00)	0.633
Total cholesterol, mmol/L	4.66 (3.99–5.43)	4.64 (3.96–5.42)	4.78 (4.04–5.54)	0.242
Triglyceride, mmol/L	1.54 (1.13–2.19)	1.46 (1.07–2.08)	1.89 (1.33–2.61)	<0.001^**^
HDL, mmol/L	1.07 (0.92–1.28)	1.09 (0.92–1.30)	1.03 (0.89–1.23)	0.025^*^
LDL, mmol/L	3.02 (2.44–3.62)	3.01 (2.43–3.59)	3.11 (2.47–3.69)	0.600
FBG, mmol/L	10.35 (7.74–13.25)	10.00 (7.44–13.15)	11.37 (8.73–14.00)	0.003^*^
CRP, mg/L	1.56 (0.76–3.45)	1.53 (0.68–3.24)	2.19 (0.99–4.30)	0.008^*^
Fasting C–peptide, pmol/L	479.51 (343.00–629.97)	475.15 (342.25–625.71)	492.50 (344.50–645.97)	0.475
Fasting insulin, pmol/L	34.60 (23.21–50.83)	34.60 (23.32–50.34)	34.49 (23.20–53.95)	0.848
HbA1c, %	10.30 (8.33–12.00)	10.20 (8.30–11.90)	10.53 (8.60–12.48)	0.131
TyG index, value	7.86 (7.42–8.27)	7.77 (7.35–8.17)	8.11 (7.75–8.51)	<0.001^**^
CMI, value	0.79 (0.51–1.21)	0.74 (0.48–1.15)	0.97 (0.66–1.35)	<0.001^**^
Men, n (%)	383 (65.60%)	289 (63.90%)	94 (71.20%)	0.122
Smoker, n (%)	247 (42.30%)	181 (40.00%)	66 (50.00%)	0.042^*^
Hypertension, n (%)	262 (44.90%)	184 (40.70%)	78 (59.10%)	<0.001^**^
Alcohol use, n (%)	209 (35.80%)	165 (36.50%)	44 (33.30%)	0.504

Note: Data are presented as median (P25 – P75) or number (%)

CVD, cardiovascular disease; TyG, triglyceride – glucose index; CMI, cardiometabolic index; BMI, body mass index; SBP, systolic blood pressure; DBP, diastolic blood pressure; HDL, high density lipoprotein; LDL, low density lipoprotein; CRP, C – reactive protein; FBG, fasting blood glucose; HbA1c, hemoglobin A1c

*, significant difference (*P* < 0.05); **, extremely significant difference (*P* < 0.001)

### Ultrasound features of the patients

The ultrasound features of the patients are detailed in Table 2. The occurrence of IMT thickening did not significantly differ between those with CVD and those without. Nevertheless, subjects with CVD demonstrated a greater burden of carotid plaques than did those without CVD, with a greater proportion of subjects having plaques (72.70% vs. 37.40%, *P* < 0.001), multiple plaques (38.60% vs. 15.50%, *P* < 0.001), and thicker plaques [1.95 (0.00–2.68) vs. 0.00 (0.00–1.60), *P* < 0.001].

**Table 2:** Carotid ultrasound features of our study population

Variables	Overall (*n* = 584)	Non – CVD Group (*n* = 452)	CVD Group (*n* = 132)	*P* value
Mean IMT, mm	0.80 (0.70–0.90)	0.70 (0.70–0.90)	0.80 (0.70–0.90)	<0.001^**^
IMT thickening, n (%)				0.132
Yes	67 (11.50%)	47 (10.40%)	20 (15.20%)	
No	517 (88.50%)	405 (89.60%)	112 (84.80%)	
Plaque presence, n (%)				<0.001^**^
Yes	265 (45.40%)	169 (37.40%)	96 (72.70%)	
No	319 (54.60%)	283 (62.60%)	36 (27.30%)	
Plaque number, n (%)				<0.001^**^
Single	144 (24.70%)	99 (21.90%)	45 (34.10%)	
Multiple	121 (20.70%)	70 (15.50%)	51 (38.60%)	
Maximum plaque thickness, mm	2.10 (1.40–2.70)	0.00 (0.00–1.60)	1.95 (0.00–2.68)	<0.001^**^
Carotid plaque burden, value	0.00 (0.00–1.00)	0.00 (0.00–1.00)	2.00 (0.00–6.00)	<0.001^**^

Note: Data are presented as median (P25 – P75) or number (%)

CVD, cardiovascular disease; IMT, intima–media thickness

**, extremely significant difference (*P* < 0.001)

### Development of the prediction models

As demonstrated in Supplementary Figure S1, following Spearman correlation analysis, a total of 23 features were employed in the construction of the predictive models. Variance Inflation Factor (VIF) analysis further confirmed that all retained predictors had VIF values below the threshold of 10 (Supplementary Table S2), indicating the absence of severe multicollinearity. As a result, all variables were included in the subsequent feature selection process.

**Fig. 2:**
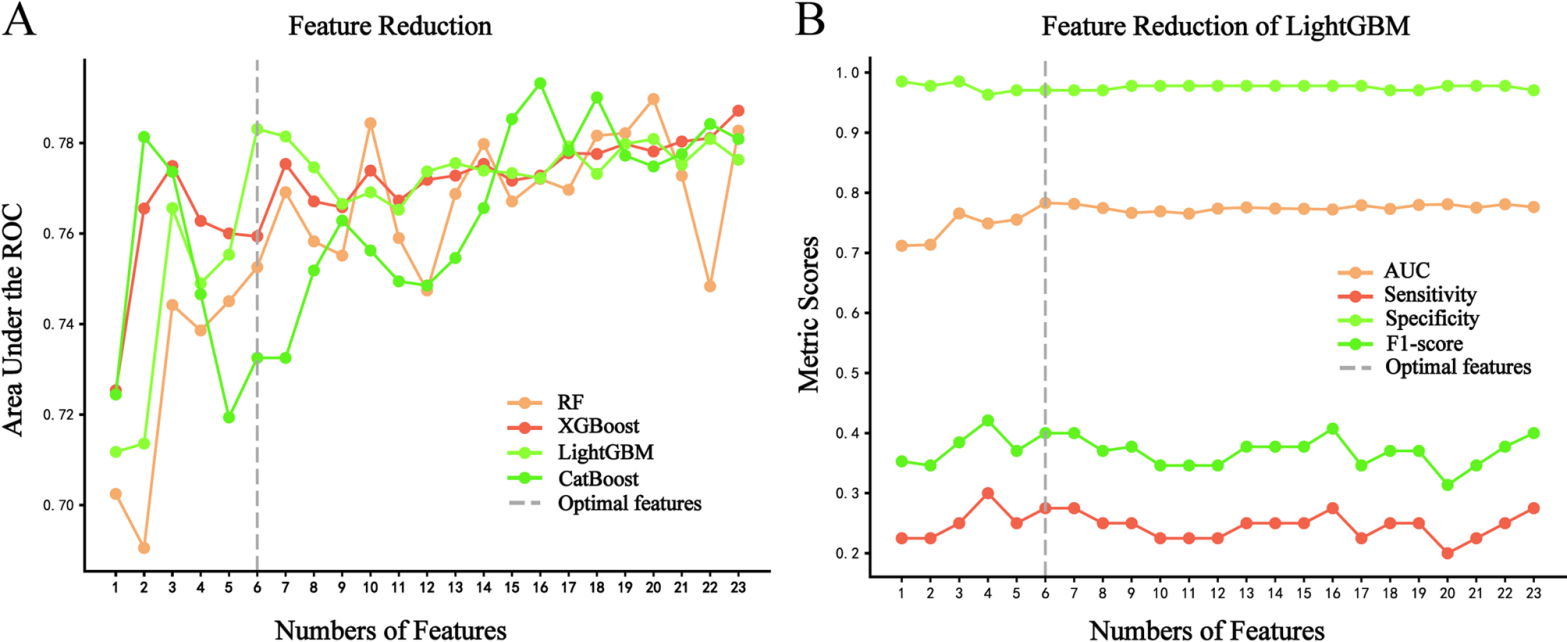
Performance of the models in predicting CVD incidence. (**A**) AUCs of different models with different numbers of features. (**B**) The AUC, sensitivity, specificity, and F1 score of the LightGBM model with various numbers of features. ROC, receiver operating characteristic curve; AUC, area under the ROC curve; RF, random forest; XGBoost, extreme gradient boosting; LightGBM, light gradient boosting machine; CatBoost, categorical boosting; CVD, cardiovascular disease

Feature selection followed a stepwise approach using SHAP rankings. Starting with 23 features, those with minimal predictive value were iteratively removed until a notable decline in model performance, measured by AUC, was observed. The four models showed varying AUCs, with LightGBM achieving stable performance using just 6 features, while other models needed more features, complicating real-world implementation (Fig. 2A). In addition, Fig. 2B and Supplementary Table S3 showed that increasing the number of features from 6 to 23 in the LightGBM model did not significantly enhance performance. Specifically, the model with all features did not outperforms those with 5 (∆AUC = 0.021, *P* = 0.304) and 6 features (∆AUC = -0.005, *P* = 0.694). However, the model with 6 features exhibited a statistically improvement over the model with 5 features (∆AUC = 0.028, *P* = 0.043). The ∆AUC indicates the difference in AUC values, with positive values indicating better performance.

After determining the feature set, model hyperparameters were fine-tuned for optimal performance using five-fold cross-validation and grid search. The performance of four ML models is compared in Supplementary Table S4, with detailed settings in Supplementary Table S5. The LightGBM model was selected based on feature selection and model evaluation results. Sensitivity analyses were conducted on this model to balance sensitivity and specificity, with a threshold of 0.2 achieving the best balance, as shown in Supplementary Table S6.

Additionally, to contextualize the model’s performance in the presence of class imbalance, we compared it with a naïve baseline classifier that always predicts the majority class (non-CVD) (Supplementary Table S7). The naïve baseline achieved an apparent accuracy of 77.3% (136/176) solely due to the skewed class distribution, but its sensitivity was 0%, indicating that no CVD cases were detected. By contrast, our LightGBM model maintained substantially higher sensitivity (65.0%), a clinically meaningful AUC of 0.772, and reasonable specificity (71.3%), demonstrating that it could provide added value by correctly identifying high-risk patients who would otherwise be missed.

**Fig. 3:**
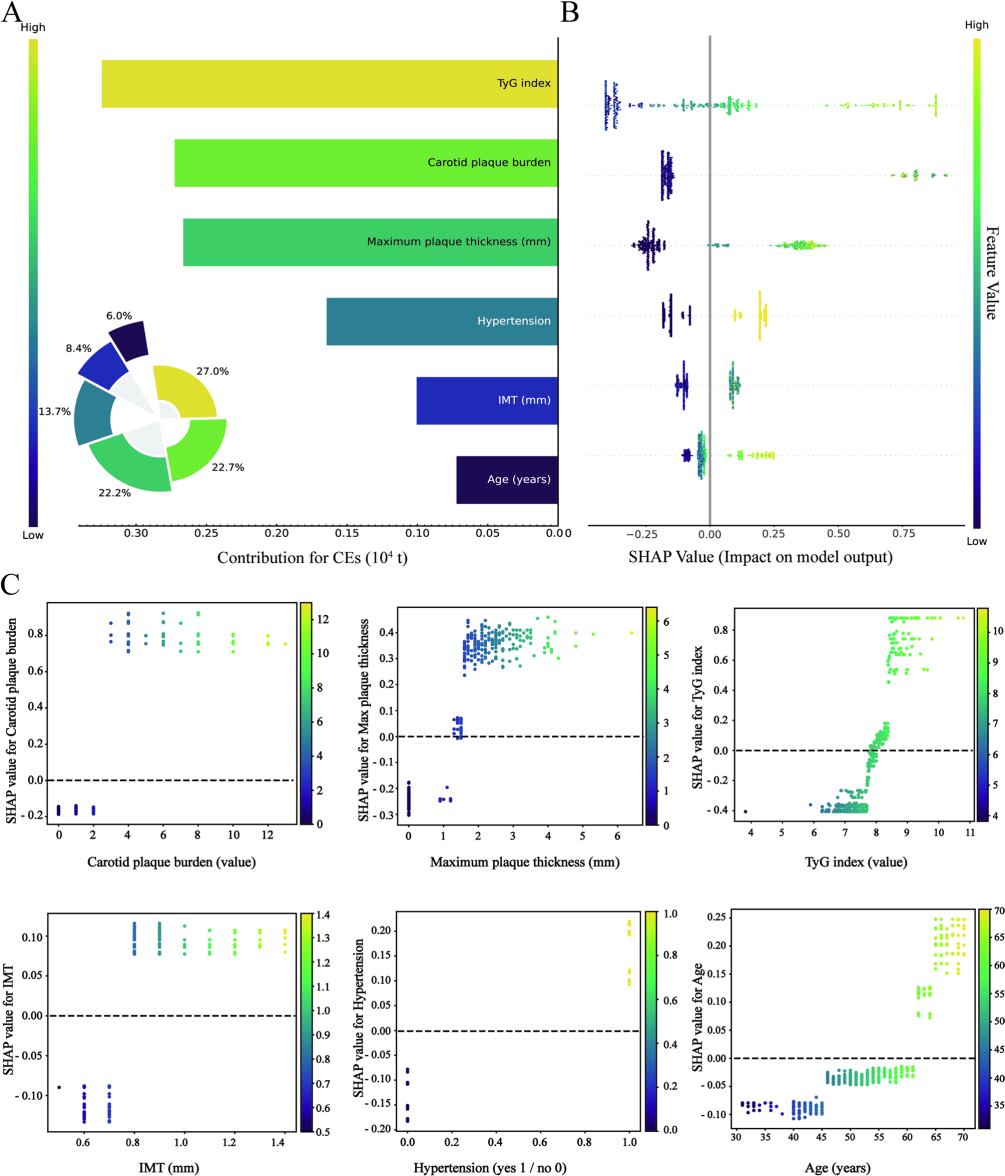
Global interpretability assessment through SHAP values. (**A**) Feature importance ranking via the SHAP bar plot. (**B**) Feature importance ranking via a SHAP dot plot. The horizontal axis represents the SHAP value magnitude, which quantifies each feature’s directional impact on CVD predictions. The features are ranked vertically by importance. (**C**) SHAP dependence plot. Dependence plots visualize the influence of individual features on CVD predictions. Threshold-exceeding SHAP values (> 0) indicate increased likelihood of CVD in the predictive algorithm. TyG, triglyceride–glucose; IMT, intima–media thickness; CVD, cardiovascular disease

Thus, the final 6-feature LightGBM model was established, which includes hypertension, age, the TyG index, carotid plaque burden, maximum plaque thickness, and IMT for further analysis (Fig. 3). Within the training cohort, the model demonstrated an AUC of 0.845, an accuracy of 0.723, a sensitivity of 0.848, a specificity of 0.687, a precision of 0.441, and an F1 score of 0.576. In the validation cohort, the model achieved an AUC of 0.772, an accuracy of 0.699, a sensitivity of 0.650, a specificity of 0.713, a precision of 0.400, and an F1 score of 0.495.

### Performance comparison with the traditional prediction model

**Fig. 4:**
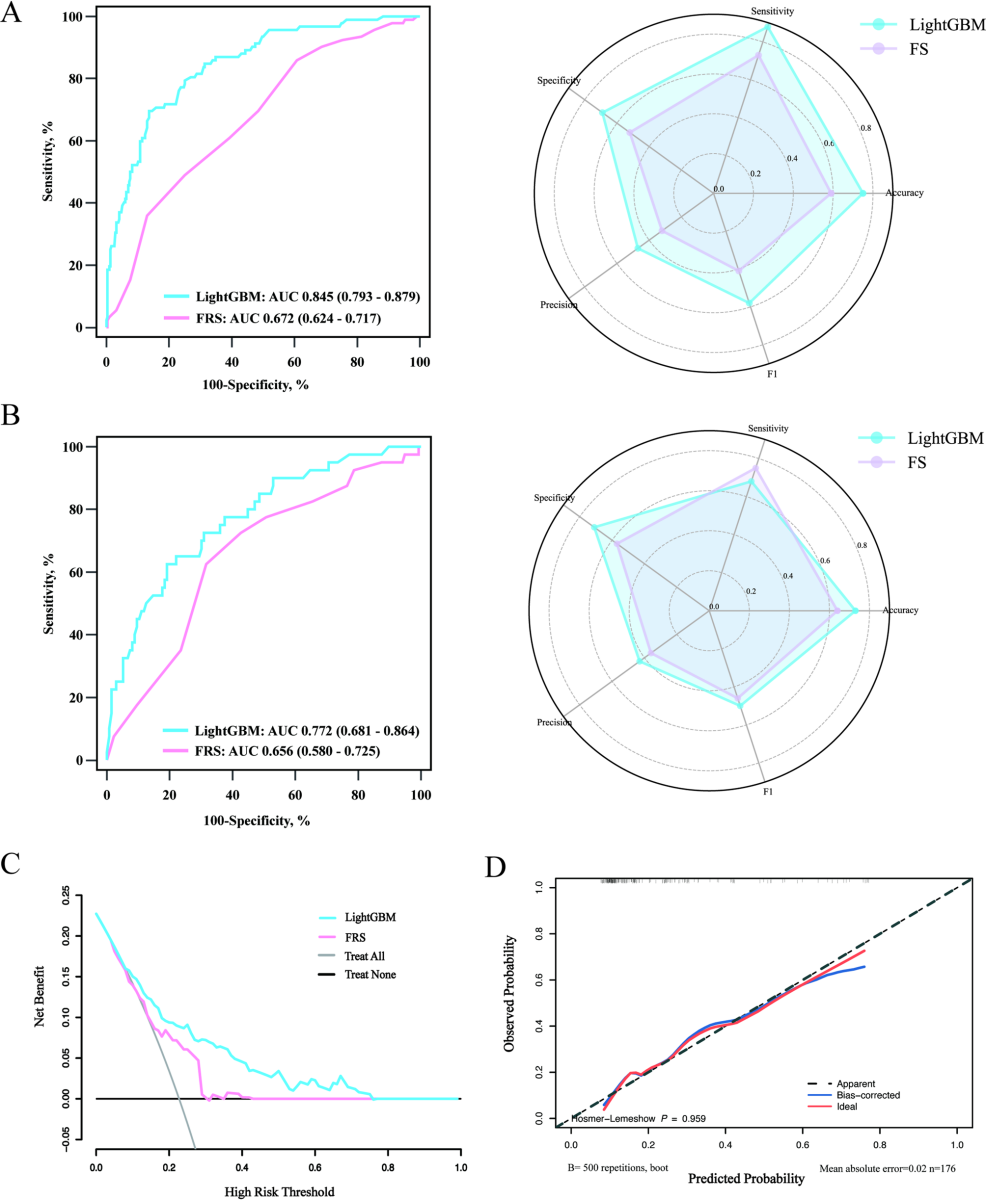
Performance comparison between the LightGBM model and the FRS for predicting 3-year cardiovascular risk in patients with new-onset type 2 diabetes. Receiver operating characteristic curves and classification metrics for the training cohort (**A**) and validation cohort (**B**). (**C**) Decision curve analysis comparing the net clinical benefit of the LightGBM model and FRS across a range of threshold probabilities. (**D**) Calibration plot of the LightGBM model in the validation cohort, demonstrating good agreement between the predicted and observed CVD risk probabilities (Hosmer–Lemeshow test: *P* = 0.959; mean absolute error = 0.02), supporting model reliability. FRS, Framingham Risk Score

We compare the performance of the LightGBM model with that of FRS, a traditional risk prediction model. Figure 4A and B present the evaluation metrics. In the training cohort, the FRS model had an AUC of 0.672, an accuracy of 0.556, a sensitivity of 0.696, a specificity of 0.516, and an F1 score of 0.414. In the validation cohort, the AUC was 0.783, the accuracy was 0.608, the precision was 0.725, the sensitivity was 0.574, the specificity was 0.333, and the F1 score was 0.457. The DeLong test revealed that the LightGBM model demonstrated superior predictive performance compared with the FRS model (*P* < 0.05 in both the training cohort and the validation cohort) (Supplementary Table S8).

In addition, to evaluate the clinical efficacy of the models, an analysis of DCA and calibration curves was performed. The findings from the DCA revealed that, compared with the FRS model, the LightGBM model exhibited better clinical utility (Fig. 4C). Moreover, the calibration curve demonstrated that the LightGBM model had favourable predictive ability (Fig. 4D).

### Model explanation and application

Given clinicians’ reliance on interpretable predictive tools, we implemented SHAP to quantify variable contributions in the final model. As depicted in Fig. 3A-B, features are ordered by mean absolute SHAP values, revealing dominant predictors of cardiovascular risk. Dependence plots (Fig. 3C) visualizing nonlinear associations between six key features and model outputs. Threshold interpretation where positive SHAP values (> 0 baseline) indicate elevated CVD probability. The horizontal dispersion in Fig. 3C’s dependence plots encodes actual measurement ranges, whereas vertical SHAP deviations quantify directional prediction impacts. Features crossing the neutral SHAP threshold (zero line) exhibit risk-enhancing effects when they exceed baseline values.

**Fig. 5:**
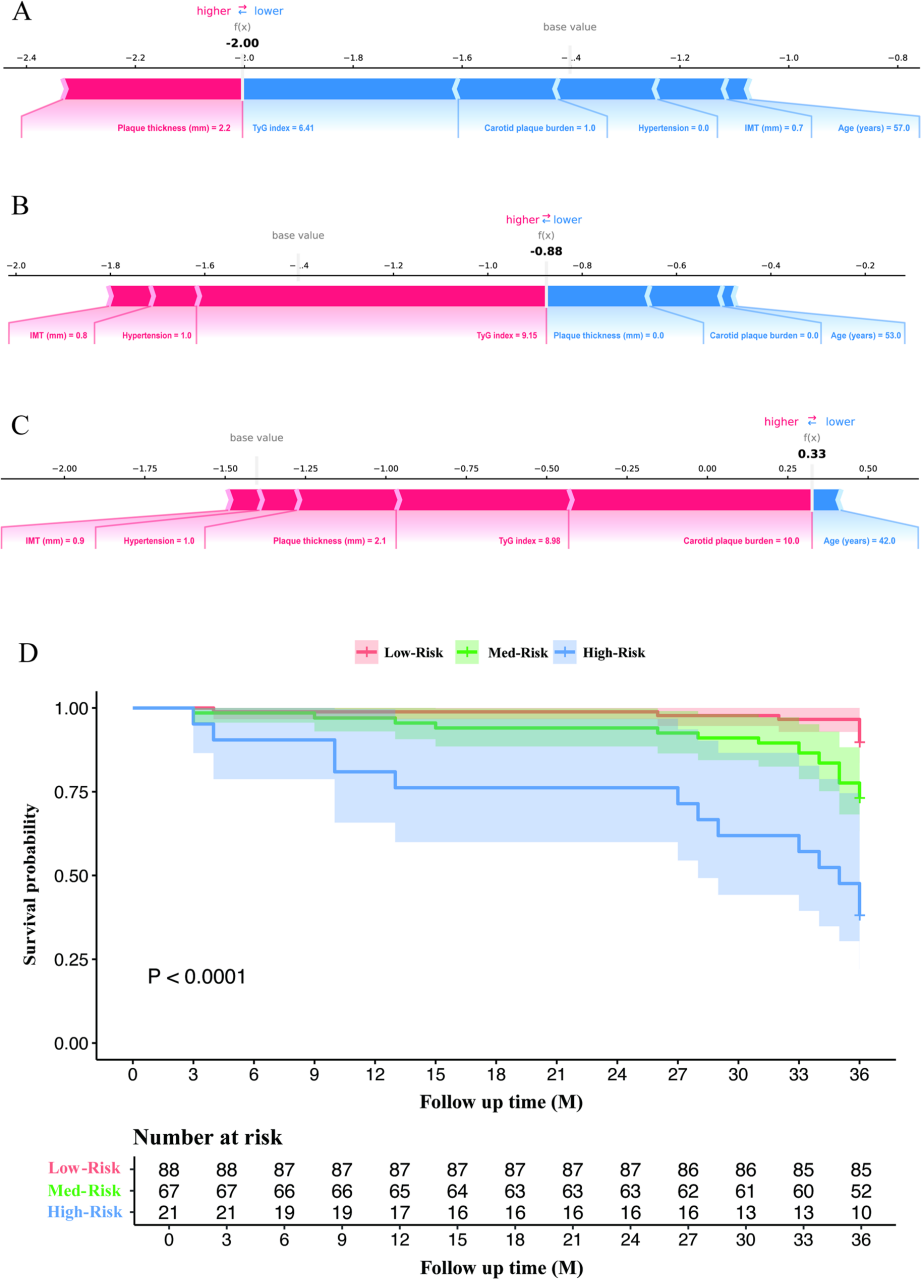
Explainable model interpretation and cardiovascular risk stratification. (**A**–**C**) SHAP force plots for three representative patients illustrating individualized prediction outputs. The blue and red bars indicate the direction and magnitude of each feature’s contribution to the model’s prediction. Patients A and B represent low- and medium-risk cases, respectively, with negative SHAP values driving the prediction below the baseline. Patient C shows high predicted risk, driven primarily by elevated carotid plaque burden, plaque thickness, IMT, and the TyG index. (**D**) Kaplan–Meier survival curves for risk stratification. Distinct difference in 3-year cardiovascular event–free survival was observed among the three groups (*P* < 0.0001), demonstrating the model’s clinical utility for risk stratification. SHAP, SHapley Additive ExPlanations; TyG, triglyceride–glucose; IMT, intima–media thickness; CVD, cardiovascular disease

Furthermore, the local explanation was used to make a particular prediction for a patient by incorporating their specific input data. Figure 5A-C illustrates the model’s assessment of patient risk levels. Specifically, in the LightGBM model, the SHAP value *f(x)* represents the logit of the predicted probability. To convert this log-odds value into a probability, the following sigmoid function was used:

$$\:\text{P}=\frac{1}{1+{e}^{-f\left(x\right)}}$$

As demonstrated in Fig. 5A, with *f(x)=*-2.00, the patient’s three-year CVD risk probability can be calculated to be approximately 11.9%. Correspondingly, as depicted in Fig. 5B and C, when *f(x)=*-0.88, the patient’s 3-year CVD risk probability is approximately 29.4%, and when *f(x) =* 0.33, the probability is approximately 58.1%.

The predictive model was also used for risk stratification to verify its prospective clinical applicability, with patients being classified into low-, medium-, and high-risk groups for CVD probabilities. The cut-off values for the three risk groups were determined to be 39.20%, 49.43%, and 11.36%, respectively, via X–tile software. Notably, the risk probabilities exhibited significant disparities across the three groups (*P* < 0.001), as presented in Fig. 5D. The middle–risk group had a risk of developing CVD that was 3.812 (95% CI 1.997–7.277) times greater than that of the low–risk group, whereas the high-risk group was 14.637 times more likely to develop CVD compared to the low-risk group, with a 95% CI of 4.328 to 49.501. These results indicate that this predictive model may provide valuable insights and enhance predictive model utility in clinical practice.

### Web application for clinical utility

**Fig. 6:**
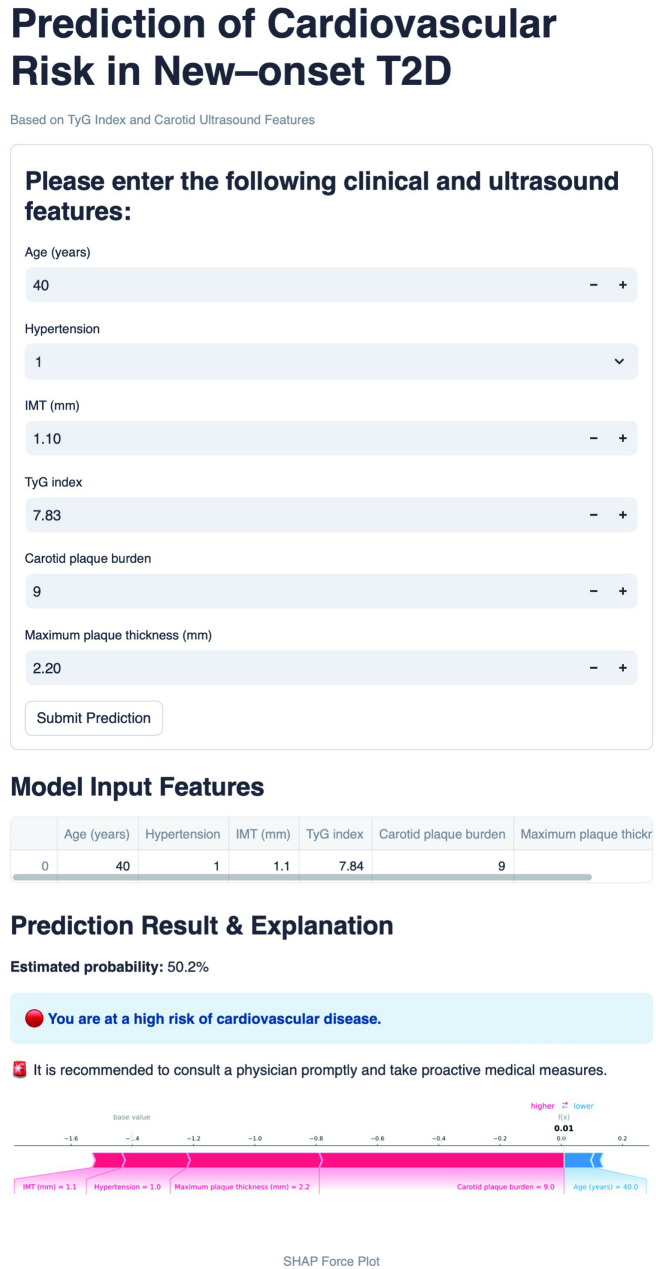
A screenshot of the web application. Input features, including age, hypertension status, IMT, TyG index, carotid plaque burden, and plaque thickness, are included. The model estimates a 50.2% probability of cardiovascular disease, categorizing the individual as high risk. A SHAP force plot provides model interpretability, visually illustrating each feature’s contribution to the prediction. A clinical recommendation is automatically generated, encouraging physician consultation and medical intervention. IMT, intima–media thickness; TyG, triglyceride–glucose; SHAP, SHapley Additive exPlanations

The prediction model was implemented as a user-friendly web-based platform (Fig. 6). By entering the values of the six essential features, the application autonomously calculates personalized 3-year CVD risk probabilities for each patient. An interactive force plot is dynamically generated for each case to demonstrate the impact of the input features on the risk prediction outcomes. In addition, the platform’s utility is further enhanced through the integration of risk-tiered suggestions. The web application can be accessed at https://prediction-model-for-cvd-in-t2d.streamlit.app/.

## Discussion

To date, few studies have applied ML models to predict cardiovascular risk in patients with new-onset T2D via integrated clinical and ultrasound data [23, 24]. Our study specifically targets this population as the initial disease burden in newly diagnosed T2D patients is often not severe, potentially resulting in their oversight of CVD prevention measures. The early identification of elevated cardiovascular risk is crucial for guiding timely intervention and preventing adverse events. In our study, an explainable LightGBM model was developed to predict 3-year CVD risk in this population, and the key findings include the following: (1) The LightGBM model achieved robust performance and stratified patients into low-, medium-, and high-risk groups, supporting its value in clinical risk management. (2) SHAP analysis identified the TyG index, carotid plaque burden, maximum plaque thickness, IMT, hypertension, and age as the most influential features, offering interpretability and actionable insights. (3) A user-friendly web application was created to enable real-time, individualized risk assessment, enhancing the model’s accessibility and clinical utility.

Notably, while increasing the number of features in prediction models may improve their predictive ability, an excessive number of features could hinder their clinical utility. Furthermore, the incorporation of noncausal features may reduce the accuracy of the models [25]. In our study, we employed a combination of recursive feature elimination (RFE) and SHAP value-based feature ranking for feature selection. Traditional RFE selects features by recursively ranking their importance and setting a step size to determine the minimum number to retain. Our method incorporates SHAP values to evaluate each feature’s contribution to model predictions, offering a more interpretable and data-driven approach. This enhances the robustness of feature selection by providing clearer insights into feature relevance, which traditional methods might miss [26].

In our study, a 0.2 threshold was set to enhance early-stage detection sensitivity, prioritizing high-risk patient identification. We found that 0.2 provided the optimal balance between sensitivity and specificity. At higher thresholds (0.3–0.5), specificity increased but sensitivity decreased markedly, which could lead to under-identification of at-risk patients. Conversely, a lower threshold (0.1) maximized sensitivity but resulted in very low specificity, producing many false positives. Therefore, a threshold of 0.2 may represent an optimal compromise [27, 28], consistent with our study’s objective of early detection while maintaining reasonable predictive performance.

In the final iteration of our algorithm, six features, namely, hypertension, age, the TyG index, carotid plaque burden, maximum plaque thickness, and IMT, were selected for model development. As presented in the results, this straightforward model could be used as a convenient tool for facilitating clinical decision–making in newly diagnosed T2D patients. Conventional risk factors for developing CVD, including sex and smoking status [29], were not incorporated into the model because the study focused on younger patients with newly diagnosed T2D and fewer comorbidities. The TyG index, which involves the multiplication of fasting glucose and triglyceride levels, is recognized as a trustworthy indicator for predicting the risk of developing CVD [30]. In our study, we identified the TyG index as the most important clinical variable incorporated in model development. Although previous research has suggested that the CMI may predict CVD development in individuals with diabetes [21], it was not incorporated into our prediction model. This exclusion can be attributed to the observation that, while CMI can indicate obesity and dyslipidaemia, its predictive power for diabetes risk is inferior to that of traditional lipid markers. The selection of the TyG index over conventional lipid markers, such as LDL and HDL cholesterol concentrations, can be justified by its comprehensive nature. The TyG index encompasses more than merely cholesterol or triglyceride levels and is strongly correlated with insulin resistance and subclinical atherosclerotic changes [31].

Carotid ultrasound examination is a noninvasive procedure for assessing the risk of developing CVD in clinical practice [32, 33]. In our study, we incorporated various ultrasound features, such as IMT, plaque presence, plaque stability, maximum plaque thickness, and plaque burden, in the model feature screening process. Our findings indicated that the IMT, plaque thickness, and plaque burden were particularly significant in the development of the model. Several studies have investigated the correlation between carotid ultrasound measurements and the likelihood of cardiovascular events in individuals with diabetes [34]. The findings revealed a significant association between the carotid plaque score measured by ultrasound and an increased risk of experiencing cardiovascular events [35]. Previous studies have also identified the IMT as a potential indicator of early arteriosclerosis development [36, 37], which is similar to the results of our analysis. These results underscore the importance of incorporating carotid plaque assessment into routine CVD risk assessment for diabetic patients.

The FRS is a widely recognized and utilized method for estimating the risk of CVD in the general population. It takes into account several risk factors, including age, gender, cholesterol levels, blood pressure, smoking habits, and diabetes, to predict a person’s 10-year likelihood of developing CVD. This scoring system has been instrumental in guiding clinical decisions and public health strategies aimed at reducing the burden of cardiovascular diseases globally [22]. Despite its widespread use, the FRS has certain limitations, particularly when applied to populations or outcomes different from those it was originally developed for [38], such as individuals with diabetes. Studies have shown that the traditional risk scoring system may underestimate CVD risk in diabetic patients due to the complex interactions among glucose metabolism, vascular health, and other risk factors [39]. This limitation can lead to suboptimal risk assessment and delayed intervention in high-risk populations. In our study, we observed that our model, which incorporates additional ultrasound features and ML algorithms, provided a more accurate prediction of CVD risk in patients with new-onset T2D than did the FRS (AUC: 0.845 vs. 0.672 in the training cohort and 0.772 vs. 0.656 in the validation cohort, *P* < 0.05 for both). This improvement can be attributed to the model’s ability to capture the unique vascular changes associated with diabetes, such as carotid plaque burden and IMT, which are not fully accounted for by the FRS [40]. Additionally, ML algorithms, such as the LightGBM model used in our study, are capable of identifying complex, nonlinear relationships between features, enhancing predictive accuracy compared with traditional risk scores [41].

Owing to the heterogeneous aetiology and complex pathophysiology of CVD in patients with diabetes, managing CVD in patients with T2D remains a significant challenge [42]. Another major finding of our study is the application of the prediction model to visualize risk stratification based on the patient’s actual condition, thereby providing appropriate recommendations. An enumeration method was employed to define clinical subgroups by grouping patients based on different cut-off values and conducting statistical analyses. The cut-off yielding the smallest *P* value was deemed optimal. Our findings indicate that the ideal cut-off values for distinguishing low-, medium-, and high-risk groups were 39.20%, 49.43%, and 11.36% respectively. The medium-risk group presented a three-year CVD risk that was 3.812 times higher than for the low-risk group, whereas the high-risk group presented a three-year CVD risk that was 14.637 times higher than that of the low-risk group. The model enhances the accuracy of identifying high-risk populations in need of proactive clinical intervention.

Although the LightGBM model performed well during training, its performance in the validation cohort was comparatively modest, which suggest potential overfitting and limited generalizability. To mitigate overfitting, we employed a rigorous feature selection strategy combining recursive feature elimination and SHAP value–based ranking, optimized hyperparameters via five-fold cross-validation, and limited the final model to six clinically interpretable features. Despite some reduction in validation performance, the model retained meaningful predictive ability, outperforming the traditional Framingham Risk Score, and effectively stratified patients into low-, medium-, and high-risk groups with significant differences in CVD incidence.

Since the direct application of ML models can be difficult for clinicians [26], our final model was integrated into a web application via the Streamlit framework, facilitating the visualization of risk stratification. This transformation enables both clinicians and patients to interact with the model effortlessly, without necessitating specialized knowledge in machine learning. Concurrently, the real-time explanatory interface empowers clinicians to corroborate risk determinants with established pathophysiological mechanisms, thereby facilitating more precise diagnostic and therapeutic interventions.

The present study is subject to several limitations. Firstly, the participant cohort was sourced from a single medical facility. Further external validation in independent cohorts may help to fully assess generalizability and clinical utility of the prediction model. Secondly, while the model predicts the occurrence of CVD within a three-year timeframe, it does not provide estimates regarding the precise timing of such events. Thirdly, the ML model was specifically developed for individuals with newly diagnosed T2D, and its applicability should be assessed in populations that include individuals with other forms of diabetes.

## Conclusion

In conclusion, this study developed an explainable cardiovascular risk prediction model for patients with new-onset T2D by combining both clinical and ultrasound imaging data. Using a streamlined set of six features—hypertension, age, the TyG index, carotid plaque burden, maximum plaque thickness, and IMT, the LightGBM model demonstrated superior predictive performance compared with traditional tools. Additionally, the model enables clinically meaningful risk stratification and was deployed as a user-friendly web application for real-time use. These findings underscore the potential of combining ultrasound imaging and ML to enhance early CVD risk detection and personalized intervention in diabetes care.

## Supplementary Information

Below is the link to the electronic supplementary material.

Supplementary Material 1

## Data Availability

The datasets used during the current study are available from the corresponding author (XTL) on reasonable request.

## References

[1] Harreiter J, Roden M. [Diabetes mellitus: definition, classification, diagnosis, screening and prevention (Update 2023)]. Wien Klin Wochenschr. 2023;135(Suppl 1):7–17. 37101021 10.1007/s00508-022-02122-y PMC10133036

[2] Nabrdalik K, Kwiendacz H, Drożdż K, Irlik K, Hendel M, Wijata AM, Nalepa J, Correa E, Hajzler W, Janota O, et al. Machine learning predicts cardiovascular events in patients with diabetes: the Silesia Diabetes-Heart project. Curr Probl Cardiol. 2023;48(7):101694. 36921649 10.1016/j.cpcardiol.2023.101694

[3] Jiang ZZ, Zhu JB, Shen HL, Zhao SS, Tang YY, Tang SQ, Liu XT, Jiang TA. A high Triglyceride-Glucose index value is associated with an increased risk of carotid plaque burden in subjects with prediabetes and New-Onset type 2 diabetes: A Real-World study. Front Cardiovasc Med. 2022;9:832491. 35310963 10.3389/fcvm.2022.832491 PMC8927542

[4] Cosentino F, Grant PJ, Aboyans V, Bailey CJ, Ceriello A, Delgado V, Federici M, Filippatos G, Grobbee DE, Hansen TB, et al. 2019 ESC guidelines on diabetes, pre-diabetes, and cardiovascular diseases developed in collaboration with the EASD. Eur Heart J. 2020;41(2):255–323. 31497854 10.1093/eurheartj/ehz486

[5] ElSayed NA, Aleppo G, Aroda VR, Bannuru RR, Brown FM, Bruemmer D, Collins BS, Das SR, Hilliard ME, Isaacs D, et al. 10. Cardiovascular disease and risk management: standards of care in Diabetes-2023. Diabetes Care. 2023;46(Suppl 1):S158–90. 36507632 10.2337/dc23-S010 PMC9810475

[6] Chen W, Li R, Yin K, Liang J, Li H, Chen X, Sheng Z, Yu H, Mu D. Clinical feasibility of using effective atomic number maps derived from non-contrast spectral computed tomography to identify non-calcified atherosclerotic plaques: a preliminary study. Quant Imaging Med Surg. 2022;12(4):2280–7. 35371951 10.21037/qims-21-643 PMC8923841

[7] Rawshani A, Rawshani A, Franzén S, Sattar N, Eliasson B, Svensson AM, Zethelius B, Miftaraj M, McGuire DK, Rosengren A, et al. Risk Factors, Mortality, and cardiovascular outcomes in patients with type 2 diabetes. N Engl J Med. 2018;379(7):633–44. 30110583 10.1056/NEJMoa1800256

[8] Amor AJ, Serra-Mir M, Martínez-González MA, Corella D, Salas-Salvadó J, Fitó M, Estruch R, Serra-Majem L, Arós F, Babio N et al. Prediction of cardiovascular disease by the Framingham-REGICOR equation in the high-risk PREDIMED cohort: impact of the mediterranean diet across different risk strata. J Am Heart Assoc 2017, 6(3). 10.1161/JAHA.116.004803 PMC5524014 28288977

[9] Serés-Noriega T, Giménez M, Perea V, Boswell L, Viñals C, Blanco J, Vinagre I, Pané A, Esmatjes E, Conget I, et al. Use of the steno T1 risk engine identifies preclinical atherosclerosis better than use of ESC/EASD-2019 in adult subjects with type 1 diabetes at high risk. Diabetes Care. 2022;45(10):2412–21. 35944257 10.2337/dc22-0118

[10] Alaa AM, Bolton T, Di Angelantonio E, Rudd JHF, van der Schaar M. Cardiovascular disease risk prediction using automated machine learning: A prospective study of 423,604 UK biobank participants. PLoS ONE. 2019;14(5):e0213653. 31091238 10.1371/journal.pone.0213653 PMC6519796

[11] Zhu L, Huang R, Li M, Fan Q, Zhao X, Wu X, Dong F. Machine Learning-Based ultrasound radiomics for evaluating the function of transplanted kidneys. Ultrasound Med Biol. 2022;48(8):1441–52. 35599077 10.1016/j.ultrasmedbio.2022.03.007

[12] Liu HQ, Lin SY, Song YD, Mai SY, Yang YD, Chen K, Wu Z, Zhao HY. Machine learning on MRI radiomic features: identification of molecular subtype alteration in breast cancer after neoadjuvant therapy. Eur Radiol. 2023;33(4):2965–74. 36418622 10.1007/s00330-022-09264-7

[13] Zheng Y, Zhou D, Liu H, Wen M. CT-based radiomics analysis of different machine learning models for differentiating benign and malignant Parotid tumors. Eur Radiol. 2022;32(10):6953–64. 35484339 10.1007/s00330-022-08830-3

[14] Gautam N, Mueller J, Alqaisi O, Gandhi T, Malkawi A, Tarun T, Alturkmani HJ, Zulqarnain MA, Pontone G, Al’Aref SJ. Machine learning in cardiovascular risk prediction and precision preventive approaches. Curr Atheroscler Rep. 2023;25(12):1069–81. 38008807 10.1007/s11883-023-01174-3

[15] Yang S, Koo BK, Hoshino M, Lee JM, Murai T, Park J, Zhang J, Hwang D, Shin ES, Doh JH, et al. CT angiographic and plaque predictors of functionally significant coronary disease and outcome using machine learning. JACC Cardiovasc Imaging. 2021;14(3):629–41. 33248965 10.1016/j.jcmg.2020.08.025

[16] Mehta A, Rigdon J, Tattersall MC, German CA, Barringer TA 3rd, Joshi PH, Sperling LS, Budoff MJ, Bertoni A, Michos ED, et al. Association of carotid artery plaque with cardiovascular events and incident coronary artery calcium in individuals with absent coronary calcification: the MESA. Circ Cardiovasc Imaging. 2021;14(4):e011701. 10.1161/CIRCIMAGING.120.011701 PMC8058258 33827231

[17] Li H, Zhang T, Han G, Huang Z, Xiao H, Ni Y, Liu B, Lin W, Lin Y. Enhanced stroke risk prediction in hypertensive patients through deep learning integration of imaging and clinical data. BMC Med Inf Decis Mak. 2025;25(1):285. 10.1186/s12911-025-03120-6 PMC12315267 40745655

[18] Jia L, Shang S, Yang Y, Zhang J, Lin X. The synergy of serum SFRP5 levels and the TyG index in predicting coronary artery disease and prognosing major adverse cardiovascular events. Lipids Health Dis. 2023;22(1):194. 37957661 10.1186/s12944-023-01965-2 PMC10642026

[19] 2. Diagnosis and classification of diabetes: standards of care in Diabetes-2024. Diabetes Care. 2024;47(Suppl 1):S20–42. 38078589 10.2337/dc24-S002 PMC10725812

[20] He C, Wu F, Fu L, Kong L, Lu Z, Qi Y, Xu H. Improving cardiovascular risk prediction with machine learning: a focus on perivascular adipose tissue characteristics. Biomed Eng Online. 2024;23(1):77. 39098936 10.1186/s12938-024-01273-5 PMC11299393

[21] Wei Q, Cheng X, Li M, Wu K, Chen M, Zhang D. Associations between the cardiometabolic index and atherosclerotic cardiovascular disease Acorss different glucose metabolism statuses: insights from NHANES, 1999–2020. Lipids Health Dis. 2025;24(1):93. 40089751 10.1186/s12944-025-02508-7 PMC11910843

[22] Towfighi A, Markovic D, Ovbiagele B. Utility of Framingham coronary heart disease risk score for predicting cardiac risk after stroke. Stroke. 2012;43(11):2942–7. 22949478 10.1161/STROKEAHA.112.668319

[23] Singh M, Kumar A, Khanna NN, Laird JR, Nicolaides A, Faa G, Johri AM, Mantella LE, Fernandes JFE, Teji JS, et al. Artificial intelligence for cardiovascular disease risk assessment in personalised framework: a scoping review. EClinicalMedicine. 2024;73:102660. 38846068 10.1016/j.eclinm.2024.102660 PMC11154124

[24] Huang Q, Zou X, Lian Z, Zhou X, Han X, Luo Y, Chen S, Wang Y, Wu S, Ji L. Predicting cardiovascular outcomes in Chinese patients with type 2 diabetes by combining risk factor trajectories and machine learning algorithm: a cohort study. Cardiovasc Diabetol. 2025;24(1):61. 39920715 10.1186/s12933-025-02611-0 PMC11806858

[25] Li Y, Sperrin M, Ashcroft DM, van Staa TP. Consistency of variety of machine learning and statistical models in predicting clinical risks of individual patients: longitudinal cohort study using cardiovascular disease as exemplar. BMJ. 2020;371:m3919. 33148619 10.1136/bmj.m3919 PMC7610202

[26] Hu J, Xu J, Li M, Jiang Z, Mao J, Feng L, Miao K, Li H, Chen J, Bai Z, et al. Identification and validation of an explainable prediction model of acute kidney injury with prognostic implications in critically ill children: a prospective multicenter cohort study. EClinicalMedicine. 2024;68:102409. 38273888 10.1016/j.eclinm.2023.102409 PMC10809096

[27] Patel BS, Steinberg E, Pfohl SR, Shah NH. Learning decision thresholds for risk stratification models from aggregate clinician behavior. J Am Med Inf Assoc. 2021;28(10):2258–64. 10.1093/jamia/ocab159 PMC8449610 34350942

[28] Sheldrick RC, Benneyan JC, Kiss IG, Briggs-Gowan MJ, Copeland W, Carter AS. Thresholds and accuracy in screening tools for early detection of psychopathology. J Child Psychol Psychiatry. 2015;56(9):936–48. 26096036 10.1111/jcpp.12442 PMC4532658

[29] Yang L, Peng Y, Zhang Z. The predictive value of triglyceride-glucose index for assessing the severity and MACE of premature coronary artery disease. Cardiovasc J Afr. 2024;34:1–6. 38407306 10.5830/CVJA-2023-060

[30] He J, Song C, Yuan S, Bian X, Lin Z, Yang M, Dou K. Triglyceride-glucose index as a suitable non-insulin-based insulin resistance marker to predict cardiovascular events in patients undergoing complex coronary artery intervention: a large-scale cohort study. Cardiovasc Diabetol. 2024;23(1):15. 38184591 10.1186/s12933-023-02110-0 PMC10771666

[31] Tao S, Yu L, Li J, Huang L, Huang X, Zhang W, Xie Z, Tan Y, Yang D. Association between the triglyceride-glucose index and 1-year major adverse cardiovascular events in patients with coronary heart disease and hypertension. Cardiovasc Diabetol. 2023;22(1):305. 37940943 10.1186/s12933-023-02018-9 PMC10633928

[32] Hughes TM, Tanley J, Chen H, Schaich CL, Yeboah J, Espeland MA, Lima JAC, Ambale-Venkatesh B, Michos ED, Ding J, et al. Subclinical vascular composites predict clinical cardiovascular disease, stroke, and dementia: the Multi-Ethnic study of atherosclerosis (MESA). Atherosclerosis. 2024;392:117521. 38552474 10.1016/j.atherosclerosis.2024.117521 PMC11240239

[33] Yin Z, Guo J, Li R, Zhou H, Zhang X, Guan S, Tian Y, Jing L, Sun Q, Li G, et al. Common carotid artery diameter and the risk of cardiovascular disease mortality: a prospective cohort study in Northeast China. BMC Public Health. 2024;24(1):251. 38254061 10.1186/s12889-024-17749-x PMC10801967

[34] Shah AS, Dabelea D, Fino NF, Dolan LM, Wadwa RP, D’Agostino R Jr., Hamman R, Marcovina S, Daniels SR, Urbina EM. Predictors of increased carotid Intima-Media thickness in youth with type 1 diabetes: the SEARCH CVD study. Diabetes Care. 2016;39(3):418–25. 26721813 10.2337/dc15-1963 PMC4764035

[35] Chen C, Zhou L, Zhou Y, Tang Y, Huang Y, Chen M. The clinical value of carotid plaque score in patients with metabolic syndrome and cardiovascular diseases. J Diabetes Complications. 2023;37(9):108546. 37579709 10.1016/j.jdiacomp.2023.108546

[36] Arida A, Fragoulis GE, Terentes-Printzios D, Konstantonis G, Protogerou AD, Vlachopoulos C, Tektonidou M, Sfikakis PP. Progression of subclinical atherosclerosis in ankylosing spondylitis: a 10-year prospective study. Rheumatol Int. 2024;44(4):643–52. 38349401 10.1007/s00296-023-05528-7

[37] Mashaba RG, Phoswa W, Maimela E, Mokgalaboni K. Association of carotid intima-media thickness and dyslipidaemia in patients with type 2 diabetes: a protocol for systematic review and meta-analysis. BMJ Open. 2024;14(1):e079209. 38262658 10.1136/bmjopen-2023-079209 PMC10823995

[38] Adil SO, Uddin F, Musa KI, Khan A, Shakeel A, Shafique K, Islam MA. Risk assessment for cardiovascular disease using the Framingham risk score and globorisk score among newly diagnosed metabolic syndrome patients. Int J Gen Med. 2023;16:4295–305. 37753441 10.2147/IJGM.S423151 PMC10518264

[39] Joshi A, Singh H, Kalra S. The acute coronary syndrome risk in medically managed subjects with type 2 diabetes Mellitus - Is the ASCVD risk score failing here? J ASEAN Fed Endocr Soc. 2024;39(1):31–6. 38863910 10.15605/jafes.039.01.15 PMC11163319

[40] Yoshida M, Mita T, Yamamoto R, Shimizu T, Ikeda F, Ohmura C, Kanazawa A, Hirose T, Kawamori R, Watada H. Combination of the Framingham risk score and carotid intima-media thickness improves the prediction of cardiovascular events in patients with type 2 diabetes. Diabetes Care. 2012;35(1):178–80. 22028278 10.2337/dc11-1333 PMC3241317

[41] Liu W, Laranjo L, Klimis H, Chiang J, Yue J, Marschner S, Quiroz JC, Jorm L, Chow CK. Machine-learning versus traditional approaches for atherosclerotic cardiovascular risk prognostication in primary prevention cohorts: a systematic review and meta-analysis. Eur Heart J Qual Care Clin Outcomes. 2023;9(4):310–22. 36869800 10.1093/ehjqcco/qcad017 PMC10284268

[42] Weir MR. Cardiovascular risk reduction in type 2 diabetes: what the non-specialist needs to know about current guidelines. Diabetes Obes Metab. 2024;26(Suppl 5):14–24. 38987977 10.1111/dom.15764

