# Supplementary material for: Development of an explainable machine learning model for 3-year cardiovascular risk prediction in new-onset type 2 diabetes using the TyG index and ultrasound features
Source: BMC Med Inform Decis Mak. 2025 Nov 4;25:409. doi: 10.1186/s12911-025-03247-6 (PMC12584365; doi:10.1186/s12911-025-03247-6)
Supplement: Supplementary file 1 — Supplementary Material 1 [file 12911_2025_3247_MOESM1_ESM.docx]

**Development of an Explainable Machine Learning Model for 3-Year Cardiovascular Risk Prediction in New-Onset Type 2 Diabetes Patients Using the TyG Index and Ultrasound Features**

1. **Supplementary** **Figure S1.** Correlation coefficients between the selected features.
2. **Supplementary Table S1.** The proportion of missing values and imputation methods.
3. **Supplementary Table S2.** Variance Inflation Factor values of the clinical features.
4. **Supplementary Table S3.** Performance of the ML model with varied numbers of features for cardiovascular risk prediction during the feature selection process.
5. **Supplementary Table S4.** Performance of the LightGBM model with varied numbers of features for cardiovascular risk prediction.
6. **Supplementary Table S5.** Detailed hyperparameter settings for the machine learning models.
7. **Supplementary Table S6.** Performance metrics of the model at different probability thresholds (0.1–0.5) for the training and validation cohorts.
8. **Supplementary Table S7.** Comparison of our model with the naïve baseline classifier.
9. **Supplementary Table S8.** Comparison of LightGBM and FRS models.

**
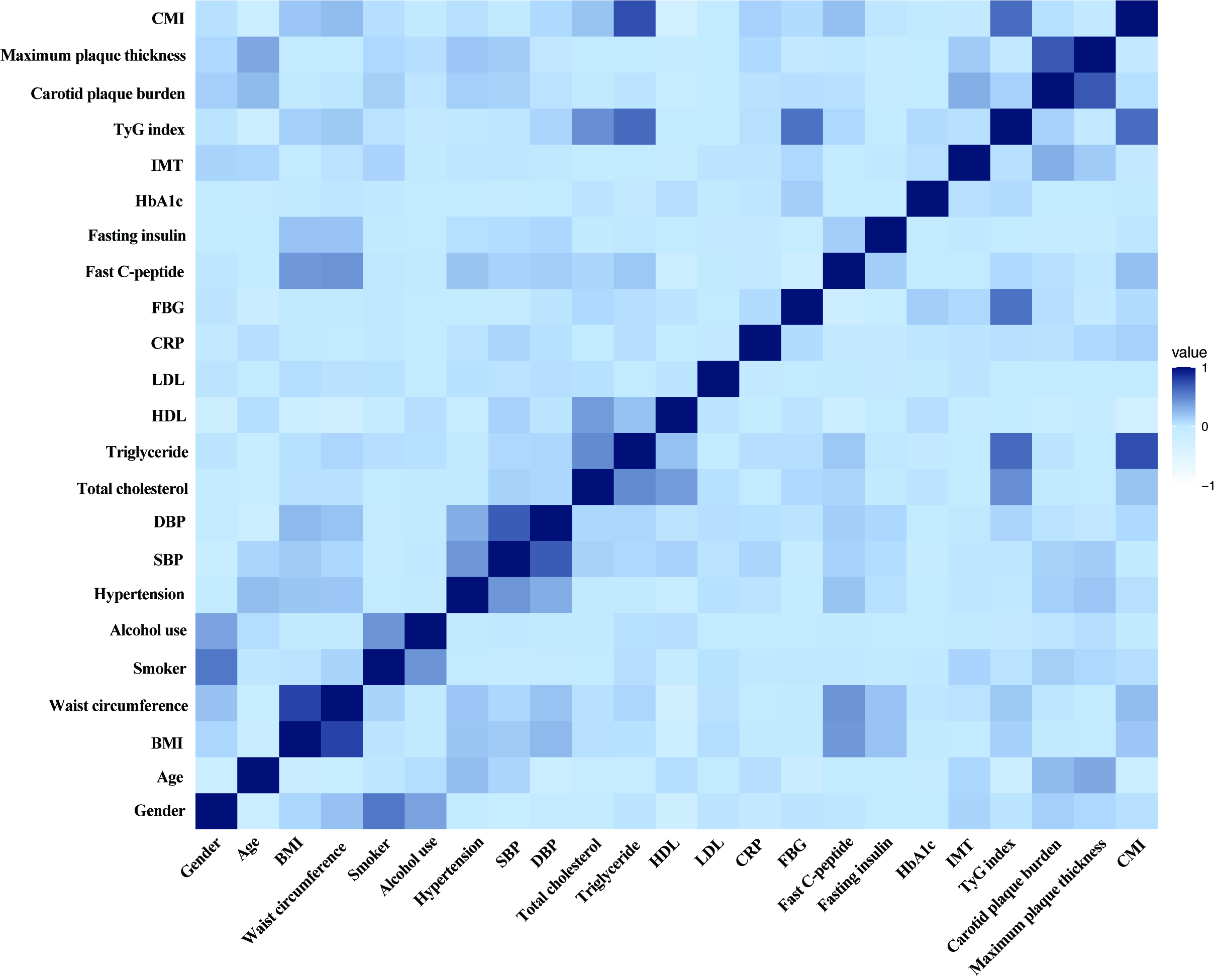
**

**1. Supplementary** **Figure S1.** Correlation coefficients between the selected features. CMI, cardiometabolic index; TyG, triglyceride–glucose index; IMT, intima–media thickness; HbA1c, hemoglobin A1c; FBG, fasting blood glucose; CRP, C – reactive protein; LDL, low density lipoprotein; HDL, high density lipoprotein; SBP, systolic blood pressure; DBP, diastolic blood pressure; BMI, body mass index.

**2. Supplementary Table S1.** The proportion of missing values and imputation methods.

| **Variable** | **Number of missing values** | **Percentage of missing values (%)** | **Imputation methods** |
| --- | --- | --- | --- |
| Smoking | 11 | 1.88 | logreg |
| Drinking | 19 | 3.25 | logreg |
| BMI | 3 | 0.51 | pmm |
| Waist circumference | 18 | 3.08 | pmm |
| Total cholesterol | 15 | 2.57 | pmm |
| Triglyceride | 16 | 2.74 | pmm |
| HDL | 17 | 2.91 | pmm |
| LDL | 14 | 2.40 | pmm |
| CRP | 39 | 6.68 | pmm |
| FBG | 4 | 0.68 | pmm |
| Fasting C–peptide | 10 | 1.71 | pmm |
| Fasting insulin | 21 | 3.60 | pmm |
| HbA1c | 29 | 4.97 | pmm |

Note: BMI, body mass index; HDL, high density lipoprotein; LDL, low density lipoprotein; CRP, C – reactive protein; FBG, fasting blood glucose; HbA1c, hemoglobin A1c; pmm, predictive mean matching; logreg, logistic regression.

**3. Supplementary Table S2.** Variance Inflation Factor values of clinical features.

| **Variable** | **VIF** |
| --- | --- |
| Sex | 1.85 |
| Age | 1.50 |
| BMI | 2.85 |
| Waist circumference | 3.02 |
| Smoking | 1.67 |
| Drinking | 1.34 |
| Hypertension history | 1.41 |
| SBP | 2.57 |
| DBP | 2.33 |
| Total cholesterol | 1.92 |
| Triglyceride | 5.47 |
| HDL | 2.50 |
| LDL | 1.04 |
| CRP | 1.08 |
| FBG | 2.74 |
| FCP | 1.42 |
| Fasting insulin | 1.08 |
| HbA1c | 1.04 |
| IMT | 1.15 |
| TyG index | 5.50 |
| Carotid plaque burden | 2.33 |
| Maximum plaque thickness | 2.25 |
| CMI | 4.84 |

Note: BMI, body mass index; SBP, systolic blood pressure; DBP, diastolic blood pressure; HDL, high-density lipoprotein; LDL, low-density lipoprotein; CRP, C-reactive protein; FBG, fastin g blood glucose; FCP, fasting C-peptide; HbA1c, hemoglobin A1c; IMT, intima-media thickness; TyG, triglyceride-glucose; CMI, cardiometabolic index; VIF, Variance Inflation Factor.

**4. Supplementary Table S3.** Performance of the ML model with varied numbers of features for cardiovascular risk prediction during the feature selection process.

| Feature numbers | AUC | Sensitivity | Specificity | F1-score |
| --- | --- | --- | --- | --- |
| 1 | 0.712 | 0.225 | 0.985 | 0.353 |
| 2 | 0.714 | 0.225 | 0.978 | 0.346 |
| 3 | 0.766 | 0.250 | 0.985 | 0.385 |
| 4 | 0.749 | 0.300 | 0.963 | 0.421 |
| 5 | 0.755 | 0.250 | 0.971 | 0.370 |
| 6 | 0.783 | 0.275 | 0.971 | 0.400 |
| 7 | 0.781 | 0.275 | 0.971 | 0.400 |
| 8 | 0.775 | 0.250 | 0.971 | 0.370 |
| 9 | 0.767 | 0.250 | 0.978 | 0.377 |
| 10 | 0.769 | 0.225 | 0.978 | 0.346 |
| 11 | 0.765 | 0.225 | 0.978 | 0.346 |
| 12 | 0.774 | 0.225 | 0.978 | 0.346 |
| 13 | 0.776 | 0.250 | 0.978 | 0.377 |
| 14 | 0.774 | 0.250 | 0.978 | 0.377 |
| 15 | 0.773 | 0.250 | 0.978 | 0.377 |
| 16 | 0.772 | 0.275 | 0.978 | 0.407 |
| 17 | 0.779 | 0.225 | 0.978 | 0.346 |
| 18 | 0.773 | 0.250 | 0.971 | 0.370 |
| 19 | 0.780 | 0.250 | 0.971 | 0.370 |
| 20 | 0.781 | 0.200 | 0.978 | 0.314 |
| 21 | 0.775 | 0.225 | 0.978 | 0.346 |
| 22 | 0.781 | 0.250 | 0.978 | 0.377 |
| 23 | 0.776 | 0.275 | 0.971 | 0.400 |

Note: AUC, area under the curve.

**5. Supplementary Table S4.** Performance comparisons of different machine learning models in the validation cohort.

|  | **AUC** | **ACC** | **PRE** | **F1–score** | **SEN** | **SPEC** | **PPV** | **NPV** |
| --- | --- | --- | --- | --- | --- | --- | --- | --- |
| RF | 0.707 | 0.659 | 0.361 | 0.464 | 0.650 | 0.662 | 0.361 | 0.865 |
| XGBoost | 0.702 | 0.648 | 0.355 | 0.466 | 0.675 | 0.640 | 0.355 | 0.870 |
| CatBoost | 0.734 | 0.676 | 0.373 | 0.467 | 0.625 | 0.691 | 0.373 | 0.862 |
| LightGBM | 0.772^*^ | 0.699 | 0.400 | 0.495 | 0.650 | 0.713 | 0.400 | 0.874 |

Note: AUC, area under the curve; ACC, accuracy; PRE, precision; SEN, sensitivity; SPEC, specificity; NPV: negative predictive value; PPV: positive predictive value; RF, random forest; XGBoost, extreme gradient boosting; LightGBM, light gradient boosting machine; CatBoost, categorical boosting.

*, significant difference (*P* < 0.05)

**6. Supplementary Table S5.** Detailed hyperparameter settings for the machine learning models.

| ML model | Detailed parameter settings |
| --- | --- |
| RF | max_features: sqrt, max_depth: 1, min_samples_leaf: 20, min_samples_split: 10, n_estimators: 10 |
| XGBoost | colsample_bytree: 0.5, gamma: 0.2, learning_rate: 0.1, max_depth: 2, n_estimators: 10, subsample: 0.5 |
| CatBoost | border_count: 32, depth: 2, iterations: 15, l2_leaf_reg: 10, learning_rate: 0.3 |
| LightGBM | colsample_bytree: 0.6, learning_rate: 0.01, 'max_depth': 2, min_child_samples: 60, n_estimators: 200, subsample: 0.6 |
|  |  |

Note: RF, random forest; XGBoost, extreme gradient boosting; LightGBM, light gradient boosting machine; CatBoost, categorical boosting; SVM, support vector machine; LR, logistic regression; MLP, multilayer perceptron; DT, decision tree; SGD, stochastic gradient descent; GaussianNB, Gaussian naïve Bayes; KNN, k-nearest neighbor.

**7. Supplementary Table S6.** Performance metrics of the model at different probability thresholds (0.1–0.5) for the training and validation cohorts.

| **Dataset** | **Probability Threshold** | **AUC** | **ACC** | **PRE** | **F1–score** | **SEN** | **SPEC** | **PPV** | **NPV** |
| --- | --- | --- | --- | --- | --- | --- | --- | --- | --- |
| Training cohort | 0.1 | 0.845 | 0.390 | 0.268 | 0.422 | 0.989 | 0.215 | 0.268 | 0.986 |
| Training cohort | 0.2 | 0.845 | 0.723 | 0.441 | 0.580 | 0.848 | 0.687 | 0.441 | 0.939 |
| Training cohort | 0.3 | 0.845 | 0.819 | 0.594 | 0.606 | 0.62 | 0.877 | 0.594 | 0.888 |
| Training cohort | 0.4 | 0.845 | 0.824 | 0.667 | 0.526 | 0.435 | 0.937 | 0.667 | 0.851 |
| Training cohort | 0.5 | 0.845 | 0.826 | 0.756 | 0.466 | 0.337 | 0.968 | 0.756 | 0.834 |
|  |  |  |  |  |  |  |  |  |  |
| Validation cohort | 0.1 | 0.772 | 0.352 | 0.257 | 0.406 | 0.975 | 0.169 | 0.257 | 0.958 |
| Validation cohort | 0.2 | 0.772 | 0.699 | 0.400 | 0.495 | 0.650 | 0.713 | 0.400 | 0.874 |
| Validation cohort | 0.3 | 0.772 | 0.795 | 0.559 | 0.514 | 0.475 | 0.89 | 0.559 | 0.852 |
| Validation cohort | 0.4 | 0.772 | 0.795 | 0.591 | 0.419 | 0.325 | 0.934 | 0.591 | 0.825 |
| Validation cohort | 0.5 | 0.772 | 0.807 | 0.750 | 0.346 | 0.225 | 0.978 | 0.750 | 0.811 |

Note: AUC, area under the curve; ACC, accuracy; PRE, precision; SEN, sensitivity; SPEC, specificity; NPV: negative predictive value.

**8. Supplementary Table S7.** Comparison of our model with the naïve baseline classifier.

| **Model** | **AUC** | **ACC** | **SEN** | **SPEC** | **PRE** | **F1 score** | **NPV** | **Confusion Matrix** |
| --- | --- | --- | --- | --- | --- | --- | --- | --- |
| Naïve baseline (always predict non-CVD) | 0.500 | 77.3% (136/176) | 0.0% (0/40) | 100% (136/136) | – | – | 77.3% | 136 / 0  40 / 0 |
| Our model | 0.772 | 69.9% (123/176) | 65.0% (26/40) | 71.3% (97/136) | 40.0% (26/65) | 49.5% | 87.4% | 97 / 39  14 / 26 |

Note: AUC, area under the curve; ACC, accuracy; PRE, precision; SEN, sensitivity; SPEC, specificity; NPV: negative predictive value.

**9. Supplementary Table S8.** Comparison of LightGBM and FRS models.

| Comparison |  | Training cohort | |  | Validation cohort | | |
| --- | --- | --- | --- | --- | --- | --- | --- |
|  | AUC | 95% *CI* | *P* value |  | AUC | 95% *CI* | *P* value |
| LightGBM vs FRS | 0.845 | 0.793-0.879 | < 0.0001^**^ |  | 0.772 | 0.681-0.864 | 0.0424^*^ |
|  | 0.672 | 0.624-0.717 |  |  | 0.656 | 0.580-0.725 |  |

Note: AUC, area under the curve; FRS, Framingham Risk Score; CI, confidence interval.

*, significant difference (*P* < 0.05); **, extremely significant difference (*P* < 0.001).
